# Iron uptake of etioplasts is independent from photosynthesis but applies the reduction-based strategy

**DOI:** 10.3389/fpls.2023.1227811

**Published:** 2023-08-11

**Authors:** Máté Sági-Kazár, Éva Sárvári, Barnabás Cseh, Levente Illés, Zoltán May, Csaba Hegedűs, Attila Barócsi, Sándor Lenk, Katalin Solymosi, Ádám Solti

**Affiliations:** ^1^ Department of Plant Physiology and Molecular Plant Biology, Institute of Biology, ELTE Eötvös Loránd University, Budapest, Hungary; ^2^ Doctoral School of Biology, Institute of Biology, ELTE Eötvös Loránd University, Budapest, Hungary; ^3^ Department of Plant Physiology, Umeå Plant Science Centre, Umeå University, Umeå, Sweden; ^4^ Department of Atomic Physics, Budapest University of Technology and Economics, Budapest, Hungary; ^5^ Institute of Materials and Environmental Chemistry, Research Centre for Natural Sciences, Eötvös Loránd Research Network, Budapest, Hungary; ^6^ Department of Plant Anatomy, Institute of Biology, ELTE Eötvös Loránd University, Budapest, Hungary

**Keywords:** chloroplast, ferric chelate reductase, Blue Native polyacrylamide gel electrophoresis, thylakoid, x-ray fluorescence imaging

## Abstract

**Introduction:**

Iron (Fe) is one of themost important cofactors in the photosynthetic apparatus, and its uptake by chloroplasts has also been associated with the operation of the photosynthetic electron transport chain during reduction-based plastidial Fe uptake. Therefore, plastidial Fe uptake was considered not to be operational in the absence of the photosynthetic activity. Nevertheless, Fe is also required for enzymatic functions unrelated to photosynthesis, highlighting the importance of Fe acquisition by non-photosynthetic plastids. Yet, it remains unclear how these plastids acquire Fe in the absence of photosynthetic function. Furthermore, plastids of etiolated tissues should already possess the ability to acquire Fe, since the biosynthesis of thylakoid membrane complexes requires a massive amount of readily available Fe. Thus, we aimed to investigate whether the reduction-based plastidial Fe uptake solely relies on the functioning photosynthetic apparatus.

**Methods:**

In our combined structure, iron content and transcript amount analysis studies, we used Savoy cabbage plant as a model, which develops natural etiolation in the inner leaves of the heads due to the shading of the outer leaf layers.

**Results:**

Foliar and plastidial Fe content of Savoy cabbage leaves decreased towards the inner leaf layers. The leaves of the innermost leaf layers proved to be etiolated, containing etioplasts that lacked the photosynthetic machinery and thus were photosynthetically inactive. However, we discovered that these etioplasts contained, and were able to take up, Fe. Although the relative transcript abundance of genes associated with plastidial Fe uptake and homeostasis decreased towards the inner leaf layers, both ferric chelate reductase FRO7 transcripts and activity were detected in the innermost leaf layer. Additionally, a significant NADP(H) pool and NAD(P)H dehydrogenase activity was detected in the etioplasts of the innermost leaf layer, indicating the presence of the reducing capacity that likely supports the reduction-based Fe uptake of etioplasts.

**Discussion:**

Based on these findings, the reduction-based plastidial Fe acquisition should not be considered exclusively dependent on the photosynthetic functions.

## 1 Introduction

Iron (Fe) plays a crucial role as a cofactor in various redox enzymes particularly in electron transport chains of mitochondria and plastids, making it one of the transition metals that are essential for life. In plant cells, chloroplasts require the highest amount of Fe, with 22 Fe nuclei necessary to operate a single linear electron transport chain in the photosynthetic apparatus; thus, chloroplasts represent the major sink of Fe in photosynthetically active cells (Hantzis et al., 2018; Schmidt et al., 2020). Fe incorporation into the Fe-containing proteins of photosynthetic machinery primarily occurs through heme groups and Fe-S clusters (Solti et al., 2012; Hantzis et al., 2018; Sági-Kazár et al., 2022); nevertheless, a minority of Fe in the chloroplasts is ligated by amino acid residues of enzymes as non-heme Fe ions.

As leaves develop in association with the division of plastids and the development of the photosynthetic apparatus, Fe content increases in the leaves as well as in the chloroplasts (Pham et al., 2020). It is generally accepted that the Fe source of the chloroplasts is the Fe pool of the cytoplasm of mesophyll cells (López-Millán et al., 2016; Vigani et al., 2019; Sági-Kazár et al., 2022). Chloroplasts of dicot models—pea (*Pisum sativum*), *Arabidopsis thaliana*, oilseed rape (*Brassica napus*), and sugar beet (*Beta vulgaris*)—were previously shown to operate a reduction-based Fe uptake system (for review, see Vigani et al., 2019 and Sági-Kazár et al., 2022). Under unstressed conditions, excess Fe accumulation in the chloroplasts is prevented by a cutoff in chloroplast Fe uptake (Solti et al., 2012). Since the expression, the substrate affinity, and the enzyme activity of oilseed rape chloroplast Ferric Reductase Oxidase 7 (FRO7) were decreased under non-toxic, but high (“supraoptimal”) Fe supply, this enzyme was suggested to be a key element in the regulation of plastidial Fe content (Sági-Kazár et al., 2021). The chloroplast outer envelope membrane does not seem to be a barrier for Fe complexes, and chloroplast FRO (cFRO) was suggested to be responsible for the substrate preference of the chloroplast Fe uptake (Bughio et al., 1997; Solti et al., 2012; Müller et al., 2019). The cFRO enzyme is localised in the chloroplast inner envelope membrane and utilises NADPH to reduce Fe complexes (Jeong et al., 2008; Solti et al., 2014a); thus, it links the plastidial Fe uptake to the operation of the photosynthetic electron transport chain (Bughio et al., 1997; Solti et al., 2012). Jeong et al. (2008) showed that *fro7* knockout mutation leads to a heavily decreased Fe content in chloroplasts. Nevertheless, photoreduction of ferric Fe compounds, such as Fe(III)-citrate, could support the reduction-based Fe uptake of chloroplasts (Jeong et al., 2008; Gracheva et al., 2022). Although the substrate preference of cFRO/FRO7 has not been tested so far, chloroplasts of oilseed rape were shown to prefer utilising Fe(III)-citrate 1:1 stoichiometry complexes in the Fe uptake (Müller et al., 2019).

Multiple Fe transport proteins were identified to target the plastid envelope membrane(s) (Sági-Kazár et al., 2022), but the precise localisation of these proteins in the outer versus in the inner envelope membranes is often unclear. It is likely that Fe(II) liberated by cFRO/FRO7 is transported across the chloroplast inner envelope membrane by Permease in Chloroplast 1 (PIC1) (Duy et al., 2007). PIC1 was suggested to collaborate with metal transport-related Ni/Co transporter (NiCo) in the Fe transport machinery based on co-expression data in a *PIC1* overexpression experiment (Duy et al., 2011). However, developmental and Fe nutrition scale analyses contradicted the exclusive collaboration between PIC1 and NiCo (Pham et al., 2020). The Mitoferrin-Like 1 (MFL1) transporter might represent a parallel or contributing member in the Fe(II) transport across the chloroplast inner envelope membrane (Tarantino et al., 2011). Moreover, *MFL1* was also shown to be suppressed in Fe-deficient wheat (*Triticum aestivum*) leaves (Hua et al., 2022), as was *PIC1* in Fe-deficient oilseed rape leaves (Pham et al., 2020). In consequence, the reduction-based uptake of Fe by chloroplasts is suppressed under Fe-deficient conditions.

Yellow Stripe-like (YSL) 4 and 6, which transport Fe-nicotianamine (NA), target the (pro)plastids in the cotyledons of Arabidopsis seedlings (Divol et al., 2013), but *YSL4* is also barely expressed in leaves of oilseed rape (Müller et al., 2019). Moreover, Conte et al. (2013) showed that YSL4/6 are in fact primarily localised in the tonoplast and thus rather involved in cellular Fe storage and redistribution. Ferroportin 3/Iron Regulated Transporter 3/Multiple Antibiotic Resistance 1 (FPN3/IREG3/MAR1) is a dual targeted protein of chloroplasts and mitochondria (Conte et al., 2009; Kim et al., 2021). Although it can transport aminoglycoside antibiotics (Conte et al., 2009; Rahman et al., 2022) and was proposed to have a role in the Fe homeostasis of plastids (Conte et al., 2009), recent data indicate that it rather has a function in the Fe unloading from mitochondria and plastids to prevent toxic Fe accumulation (Kim et al., 2021). There is no clear evidence that Fe–NA complexes would be involved in Fe loading to chloroplasts; however, NA was suggested to play a role in maintaining Fe solubility, as the disruption of NA biosynthesis leads to Fe-phosphate precipitation in plastids as observed with the tomato *chloronerva* mutant (Becker et al., 1995; Liu et al., 1998). Most likely, NA is involved in Fe ligation once Fe is liberated (Curie and Briat, 2003). On the other hand, Fe that is taken up into the chloroplast stroma is immediately subjected to the formation of Fe-S clusters and heme groups (Solti et al., 2012). During chloroplast development, the induction of the plastidial SUF system suggests the importance of Fe-S biogenesis (Sági-Kazár et al., 2021), where the photosynthetic apparatus requires the majority of Fe-S clusters (Connorton et al., 2017). The *Arabidopsis* NEET motif protein, anchored to the outer envelope membrane of both mitochondria and chloroplasts, was shown to take part in the transfer of Fe-S clusters towards cytosolic Fe-S proteins (Nechushtai et al., 2020; Zandalinas et al., 2020; Zandalinas et al., 2022).

In angiosperms, the biosynthesis of chlorophyll (Chl), which is crucial for the development of the photosynthetic apparatus and Chl proteins, and the differentiation of plastids into chloroplasts are dependent on light (Solymosi and Schoefs, 2010; Solymosi and Mysliwa-Kurdziel, 2021). One of the key enzymes of Chl biosynthesis, NADPH:protochlorophyllide oxidoreductase (LPOR, EC 1.3.1.33), requires light for its activity, i.e., for the reduction of protochlorophyllide (Pchlide) into chlorophyllide (Solymosi et al., 2004; Kruk, 2005; Gabruk and Mysliwa-Kurdziel, 2015; Erdei et al., 2016). Therefore, in the absence of light, Chl biosynthesis is inhibited, and proplastids convert into etioplasts accumulating the tubular membrane structure called prolamellar body that lacks the components of the photosynthetic machinery (Sandoval-Ibáñez et al., 2021). This etiolation syndrome commonly occurs in nature, especially in leaf buds (Solymosi and Böddi, 2006; Solymosi et al., 2006; Solymosi et al., 2012) and soil-covered stem segments (Vitányi et al., 2013; Kakuszi et al., 2016; Kakuszi et al., 2017).

Although Fe is primarily required by the biosynthesis of the photosynthetic machinery in the chloroplasts, multiple metabolic pathways and redox enzymes also require the presence of Fe and Fe cofactors. Nevertheless, the uptake of Fe by non-photosynthetic plastids has not been explored so far. In addition, the fact that Fe uptake in chloroplasts is entirely dependent on light raises questions about how Fe loading operates in plastids that develop without light exposure *ab ovo*. The inner leaf primordia of cabbage heads, which are shaded by the outer green leaves and develop in the absence of light, accumulate photoactive Chl precursors and contain non-photosynthetic plastids (Solymosi et al., 2004; Kruk, 2005; Erdei et al., 2016). As a result, cabbage heads, which represent a giant modified bud, provide a suitable model system for investigating Fe uptake in non-green plastids, as has been demonstrated in previous studies.

## 2 Materials and methods

### 2.1 Plant material

Savoy cabbage (*Brassica oleracea* var. *sabauda* L.) heads were obtained from local markets right after harvesting. For each measurement, Savoy cabbage heads of similar size and weight were selected. Sample preparation and experimental procedures were carried out in a dark room illuminated with green safelight. Savoy cabbage heads were separated, and leaves were sorted into groups (“layers”) numbered from the outermost, light-exposed leaves according to the 2/5 phyllotactic pattern of Savoy cabbage and morphology of the leaves (**Supplementary Figure 1**). Each layer consisted of four leaves that were used and evaluated together for further physiological and molecular studies (**Supplementary Figure 2**).

### 2.2 Chlorophyll *a* fluorescence induction

The status of the photosynthetic electron transport chain was assessed by Chl *a* fluorescence induction. Leaves of various degrees of etiolation were measured as in Kakuszi et al. (2016). Briefly, a PAM 101–102–103 (Heinz Waltz, Effeltrich, Germany) fluorometer was applied. Ground fluorescence (*F*_0_) was determined by turning on the measuring light (PPFD < 1 μmol m^−2^ s^−1^ modulation frequency: 1.6 kHz). Maximal fluorescence (*F*_m_) values were measured as response to a 0.7-s, 3,500 μmol m^−2^ s^−1^ PPFD flash illumination (light source: KL 1500 electronic, Schott, Mainz, Germany). The maximal efficiency of the PSII was calculated by the following equation: *F*_v_/*F*_m_ = (*F*_m_ − *F*_0_)/*F*_m_. Non-photochemical quenching related to the dissipation of the inactive photosystem II (PSII) reaction centres were calculated according to Hendrickson et al. (2005) applied according to Solti et al. (2014b): Φ_NF_ = 1 − [*F*_v_/*F*_m_)/(*F*_vM_/*F*_mM_)].

### 2.3 X-ray fluorescence imaging

Distribution of elements in the leaves was analysed by x-ray fluorescence (XRF) imaging. Leaves were dried at 60°C for 48 h, ensuring the flat surface without pressing the leaves during drying. Equivalent leaf lamina positions of different leaves from each layer were applied to an open sampler holder. XRF imaging was performed by a Horiba XGT7200V instrument equipped with an Rh x-ray tube and a silicon drift detector. Energy calibration was performed using a Cu plate according to the manufacturer’s instruction. Ranges of interest (ROIs) were measured applying an x-ray guide tube diameter of 100 μm operated at 50 kV and 1 mA in vacuum. XRF photons were detected by a silicon drift detector. Distribution of Fe, Mn, and Mg was analysed based on the characteristic Kα_1_ photons emitted at 6.40-, 5.90-, and 1.25-keV peaks. Data analysis and representation were carried out using the Hyperspy multi-dimensional data analysis package (v1.7.1; de la Peña et al., 2022) in Python 3 (3.11).

### 2.4 Quantification of element concentrations

Leaf lamina-enriched samples were established by removing the major vein regions of the leaves. Samples were dried at 85°C for 2 days. Dry weight of the samples was recorded in the air-dry stage. Samples were digested according to a three-step protocol: first in cc. H_2_O_2_ for 1 h at room temperature, then in cc. HNO_3_ for 15 min at 60°C, and finally in the same solution for 45 min at 120°C. Digested samples were filtered using MN 640 W paper (Macherey-Nagel, Düren, Germany). Element concentrations were analysed by ICP-OES (Spectro Genesis, SPECTRO, Freital, Germany) mounted with a simultaneous axial plasma spectrometer.

### 2.5 Transmission electron microscopy

Leaf pieces were fixed in 2.5% (w/V) glutaraldehyde in 70 mM Na-K phosphate (pH 7.2) buffer for 2 h in the dark as in Ounoki et al. (2021). Post-fixation was performed in 1% (w/V) OsO_4_ for 3 h, in the same buffer. After dehydration in an alcohol series, the samples were embedded in Durcupan ACM (Fluka); 60-nm ultrathin sections were cut with Reichert Jung Ultracut E microtome (Reichert-Jung AG, Vienna, Austria). The sections were stained with uranyl acetate and lead citrate. Ultrathin sections were examined in a Hitachi 7100 electron microscope (Hitachi Ltd., Tokyo, Japan) at 75 kV accelerating voltage. TEM micrographs were taken with a MegaView III camera (Soft Imaging System, Münster, Germany).

### 2.6 Chlorophyll content measurement

Leaf disks of known weight and diameter were homogenised in 80% (V/V) acetone buffered by 5 mM Tricine (pH 7.8) and centrifuged at 10,000 *×g* for 5 min at 4°C. Total Chl content in the supernatant was determined using a UV-VIS 2600 spectrophotometer (Shimadzu, Japan) according to Porra et al. (1989). To cope with residual light scattering, extinctions at *E* = 800 and 730 nm were recorded.

### 2.7 Identification of *Arabidopsis* sequences in *Brassica oleracea*

*A. thaliana* transcript and protein sequences were accessed in the NCBI database using the Araport IDs of selected targets. To identify putative homologs of *A. thaliana* sequences in *B. oleracea*, protein sequence blasting was performed in NCBI. The returned best hits and corresponding transcript accessions were used to perform reciprocal protein and nucleotide blasts against *A. thaliana* to verify our queries as genes encoding *B. oleracea* orthologues. Blast results are listed in **Supplementary Data Sheet 1**. Genes encoding *B. oleracea* orthologues of the *Arabidopsis* queries were identified as listed in **Table 1**.

**Table 1:** Oligonucleotide primers used in expression analysis. Forward (FW) and reverse (RV) primers were applied on the annealing temperature (*T*_m_) determined in preliminary studies.

Gene (NCBI accession, Arabidopsis orthologue)		Primer sequences	Product (bp)	*T*_m_ (°C)
		(5′ → 3′)		
ABCI8 (XM_013756524.1, AT4G04770.1)	FW	GGGTATCTCGGCTGGCAACT	163	58
	RV	GGCTGATGGGTTCTTAACCTGGAT		
FRO7 (XM_013756407.1, AT5G49740.1)	FW	GGTGTTCGCTAAGAAGAAGATATCG	151	57.5
	RV	GTCAAGATCCCTCATGGTATATGC		
FER1 (XM_013773172.1, AT5G01600.1)	FW	ACTCCACCCTATCGTCTCCC	143	59
	RV	TGTTCTCTGATGCCACTCTGT		
PIC1 (XM_013738218.1, AT2G15290.1)	FW	TGCGGTCACTACTCTTGC	161	59
	RV	GATGGTGGCTCTCCTCTTC		
FPN3 (XM_013757359.1, AT5G26820.1)	FW	GGCTCTTCTCAGACAATCTCC	98	59
	RV	TGCGAACTCCAGACAAACC		
MFL1 (XM_013782497.1, AT5G42130.1)	FW	CGTGCGAGTTCGGTAAGTCA	219	59
	RV	CAGCGTAGAGCCCCAAGATT		
ABCI11 (XM_013751578.1, AT5G14100.1)	FW	CCCTTGCTGGTCTTGATTGG	178	59
	RV	TAAACTGGTGGACGCTCTGC		
NEET (XM_013753856.1, AT5G51720.1)	FW	AGGAAGCAGCAGAGAATGGC	105	59
	RV	TACCACGACAGAGTCCACCA		
YSL4 (XM_013776516.1, AT5G41000.1)	FW	TCAGTCTCGTTCCACTTCG	112	57
	RV	TCGGCTCCAGAGTTGTCA		
YSL6 (XM_013756592.1, AT3G27020.1)	FW	TCTCAATCTACCCGTTGTTACC	138	59
	RV	GCAAGACCCACATAGCCAGA		
TUB4 (XM_013756763.1, AT5G44340.1)	FW	TCGATCCAGGAGATGTTCAG	148	59
	RV	ACTCTGCAACAAGATCATTCATG		
EF1α (XM_013730661.1, AT1G07940.1)	FW	CAGATCAACGAGCCAAAGAGG	120	56
	RV	CTTGAGCATACCGGTCTCAAC		
18s RNA (KT377451.1, AT3G41768.1)	FW	GCATTCGTATTTCATAGTCAGAGGTG	192	61
	RV	CGGAGTCCTAAAAGCAACATCC		

Specific PCR products were amplified for each primer pair (size given in base pairs: bp).

### 2.8 Expression analysis

The preparation of cDNA samples was performed as in Sági-Kazár et al. (2021). Briefly, total RNA was extracted from approximately 80 mg of lamina enriched leaf tissue in 0.5 ml of TRI Reagent (Sigma) using Direct-Zol™ RNA MiniPrep (Zymo Research, USA) according to manufacturer’s instructions. Pelleted nucleic acids were resolved in 30 μl of DNase/RNase-free water. RNA concentration and purity were checked by Nanodrop ND-1000 spectrophotometer (Thermo Fisher Scientific). Reverse transcription and cDNA creation were carried out using RevertAid Reverse Transcriptase (Thermo Fisher Scientific) at 42°C for 45 min using random hexamer oligonucleotides, and cDNA libraries were stored at −80°C until use.

Relative transcript analysis was carried out by quantitative real-time PCR (qPCR). As for reference genes of the qPCR studies, *18S rRNA* (KT377451.1), *TUB4* (XM_013756763.1), and *EF1α* (XM_013730661.1) coding sequences were accessed in NCBI database and tested for expression in Savoy cabbage as in Pham et al. (2020). Primer oligonucleotides are listed in **Table 1**. Specificity of primers was verified by melt curve analysis and agarose gel electrophoresis of PCR products. Efficiency of primers was estimated based on standard curve analysis using seven points of twofold serial dilutions of cDNA template. Efficiency of the PCR reaction for each gene ranged from 1.87 to 1.99. Expression analysis was performed using a StepOnePlus Real-Time PCR system (Thermo Fisher Scientific) operated with StepOneTM v.2.2.3 software. Amplification was followed by SYBR Green (Luminaris Colour HiGreen High ROX; Thermo Fisher Scientific). The qRT-PCR program was 50°C, 2 min pre-digesting, 95°C, 10 min initial denaturation, 40 cycles with the following profile: 95°C, 15 s denaturation, primer specific *T*_m_, 30 s annealing, and 72°C, 30 s elongation. Program was terminated by melt curve analysis. All cDNA samples were freshly diluted for qPCR analysis. Three technical and five biological parallels were analysed. Normalised relative expression was calculated according to Pfaffl (2001).

### 2.9 Isolation of plastids

Plastids of leaves of various degrees of etiolation were isolated as in Kakuszi et al. (2016) and Müller et al. (2019) in a dark room illuminated with green safelight. Briefly, leaves were homogenised using a Waring Blender for 8 s in an isolation buffer containing 0.4 M sorbitol, 2 mM EDTA, 50 mM HEPES, and 0.1% (w/V) Na-ascorbate (pH 7.5). The homogenate was filtered through two layers of gauze and one layer of Miracloth™ (Calbiochem-Novabiochem, San Diego, USA) and centrifuged at 4,000 *×g* for 5 min in a swing-out rotor at 4°C. Pellets were resuspended in isolation buffer, layered onto a two-step Percoll gradient [65/35% (w/V) Percoll, 0.4 M sorbitol, 2 mM EDTA, and 50 mM HEPES, pH 7.5], and pelleted at 4,800 *×g* for 8 min. Intact plastids were collected from the 65/35% gradient interface, resuspended in a washing buffer (0.4 M sorbitol and 50 mM HEPES, pH 7.5), and pelleted at 4,800 *×g*, 2 min. Pellets were resuspended in the washing buffer. Plastid densities were determined by microscopy counting in a Bürker chamber using a Nikon Optiphot-2 microscope.

### 2.10 Chlorophyll fluorescence lifetime measurements

Chl fluorescence lifetime was characterised as the decay in the Chl fluorescence in intact leaves. Isolated intact plastids were filled into a quartz cuvette (Hellma 101-10-40) placed in a commercial holder with fibre adapter (Thorlabs CVH100/M) and measured in a right-angle observation of the centre of a centrally illuminated cuvette (Lakowicz, 2006). The samples were illuminated using a fibre-coupled laser diode (PicoQuant LDH-P-C-650) emitting at 650 nm with 10-MHz repetition rates, <100-ps pulse lengths, and ca. 3-mW laser power using a PicoQuant PDL 828 Sepia II laser diode driver. The fluorescence lifetimes were accumulated with the time-correlated single photon counting (TCSPC) technique using PicoHarp 300 (PicoQuant) TCSPC electronics with a 4-ps time resolution. The emitted fluorescence photons were collected by collimating optics (Thorlabs B270) into a multimode optical fibre (Thorlabs M18L01) and detected by a single-photon detector (PicoQuant MPD-100-CTB). To eliminate all scattered light, a combination of a long-pass optical coloured glass filter (Thorlabs FGB25) and a bandpass optical interference filter (Semrock 708/75) as an emission filter was applied. The transmitted excitation was absorbed using a beam trap (Thorlabs BTC30). The instrument response function was determined to a value of 0.12 ± 0.01 ns full width at half maximum using the scattered light of the buffer without any emission filters. In order to avoid any pile-up effect, an OD1 neutral density filter (Thorlabs NE510B-A) was used in case of highly fluorescing samples. Fluorescence lifetime curves were analysed using the EasyTau2 (PicoQuant) fluorescence lifetime analysis program. A deconvolution with two exponentials was fitted, and intensity weighted average lifetimes were calculated.

### 2.11 NADP(H) pool determination

NADP(H) determination was carried out according to Wagner and Scott (1994) with slight modifications. Isolated plastids were mixed with equal volume of extraction buffer [20 mM NaHCO_3_, 100 mM Na_2_CO_3_, and 1% (m/V) Triton X-100] and centrifuged at 16,000 *×g* for 1 min at 4°C. Supernatant was added to a cycling buffer [100 mM Tris-HCl (pH 8.0), 0.5 mM Thiazolyl Blue Tetrazolium Bromide (MTT), 2 mM phenazine ethosulphate (PES), 5 mM EDTA, and 1.3 U ml^−1^ glucose-6-phosphate dehydrogenase (G6PD) enzyme in 1:8 ratio] and incubated at 37°C for 2 min. Enzyme reaction was initiated by the addition of 10 mM glucose-6-phosphate to the sample in 1:9 ratio. The operation of G6PD results in the reduction of NADP^+^ to NADPH, which reacts with MTT through PES, generating formazan. The reaction was followed by the formazan formation at 570 nm for 5 min. NADPH concentrations were calculated based on a calibration curve of NADPH standards.

### 2.12 Fe content and Fe uptake of plastids

Fe content and Fe uptake measurements were carried out based on Zelenyánszki and Solti (2023). Intact plastids were solubilised in the previous washing buffer containing 1% (m/V) sodium dodecyl sulphate (SDS) and 1% (m/V) dithiothreitol (DTT) for 30 min at room temperature. Non-solubilised material (primarily starch) was removed by centrifugation at 10,000 *×g* for 5 min. Liberated Fe content of the samples was reduced by the addition of 100 μM ascorbic acid. Reduced Fe was chelated by the addition of 300 μM bathophenanthroline disulfonate disodium salt (BPDS, Sigma), resulting in the formation of [FeBPDS_3_]^4−^ complexes. Absorbance of the complexes was determined using a UV-VIS 2600 spectrophotometer at 535 nm. The concentration of Fe was calculated with the absorption coefficient of 22.14 mM^−1^ cm^−1^ (Smith et al., 1952).

To determine the Fe uptake of the isolated plastids, the method of Solti et al. (2012) was applied. Briefly, plastid suspensions were kept on ice in darkness. Fe(III)-citrate was added to the ice-cold suspensions. To initiate Fe uptake, suspensions were warmed up to room temperature in darkness for 5 min. Fe uptake of plastid suspensions was carried out both in darkness or in 120 μmol m^−2^ s^−1^ PPFD (Philips HPI-T Plus, 250 W metal halogen lamp). Fe uptake was terminated by cooling down the suspensions on ice in darkness. Samples were centrifuged promptly at 2,500 *×g* in a swing-out rotor for 5 min. Pelleted chloroplasts were washed in 0.25 ml of the identical buffer containing 2 mM (V/V) EDTA to remove surface-associated Fe and centrifuged again. The Fe content of the suspensions was determined as described before applying the BPDS-based method. Fe uptake of the plastids was calculated as the difference between the Fe content of the samples taken before and after the incubation.

### 2.13 Isolation of plastid envelope membranes

Envelope membranes of plastids were purified as in Solti et al. (2014a) and in Sági-Kazár et al. (2021) with slight modifications. Isolated plastids were transferred into TE buffer (10 mM Tris-HCl, pH 7.8, and 2 mM EDTA) containing 0.6 M sucrose and broken using three freeze/thaw cycles (−20/0°C) then diluted three times in TE buffer and further incubated on ice for 60 min. Thylakoids were removed with 6,000 *×g*, 20 min centrifugation at 4°C. Membranes from the supernatant were pelleted by high-speed centrifugation at 22,000 rpm, 60 min, 4°C in a Sw40Ti rotor operated by a Beckman L7 ultracentrifuge (Beckman Coulter). The pellet was resuspended in TE buffer containing 0.2 M sucrose and layered on a stepwise sucrose gradient (1/0.8/0.45 M sucrose in TE buffer). Gradient ultracentrifugation was performed at 35,000 rpm, 120 min at 4°C. Inner envelope membrane (IE) vesicles were collected from the 1/0.8 M gradient interface, diluted with TE buffer and pelleted at 25,000 rpm, 60 min at 4°C. Pellets were resuspended in TE buffer and stored at −80°C until use.

The identity of plastidial IE samples was checked by immunoblotting. Membrane fractions were solubilised in 62.5 mM Tris-HCl, pH 6.8, 2% (m/V) SDS, 2% (m/V) DTT, 10% (m/V) glycerol, and 0.001% (m/V) bromophenol blue at room temperature for 30 min. Proteins were separated by polyacrylamide gel electrophoresis (PAGE) according to Laemmli (1970) using 10%–18% gradient gels in a MiniProtean apparatus (Bio-Rad) on 20 mA constant current at 6°C. Protein concentration of samples was determined by comparative densitometry according to Sárvári et al. (2022). Western blotting was performed against 37 kDa Inner Envelope Protein 37 (IEP37, IE marker) and Light Harvesting Complex apoproteins (apoLHC, a thylakoid marker). Membrane proteins separated by SDS-PAGE were transferred to Amersham™ Protran™ premium 0.2 μm NC (Ge Healthcare, IL, USA) nitrocellulose membranes in 25 mM Tris, pH 8.3, 192 mM glycine, 20% (V/V) methanol, and 0.02% SDS at 4°C using 90 V constant voltage (<0.4 A) for 3 h. Membranes were decorated with rabbit polyclonal antibodies against apoLHCII (a gift from Dr. Udo Johanningmeier, Bochum University, Germany) and IEP37 (a gift from Prof. Katrin Philippar, Saarbrücken University, Germany). Antibodies were dissolved in 20 mM Tris-HCl, pH 7.5, 0.15 M NaCl, and 1% (m/V) gelatine. Horseradish peroxidase (HRP)-conjugated goat anti-rabbit IgG (Bio-Rad) was used according to the manufacturer’s instructions. Blots were scanned using an Epson Perfection V750 PRO scanner and analysed in Phoretix image analysis software (Phoretix International, Newcastle- upon-Tyne, UK).

### 2.14 Separation of the main plastid complexes by Blue Native PAGE

The separation of main complexes present in the plastids was performed by Blue Native (BN) PAGE (Sárvári et al., 2022) using 4.5%–12% (w/V) gradient gels of 1.5 mm thickness (Mini-Protean, Bio-Rad). Samples of similar protein content were solubilised with 1% (w/V) n-dodecyl-*β*-D-maltoside (*β*-DM, Sigma) plus 1% (w/V) digitonin (Serva) on ice for 30 min. BN PAGE was carried out at 6°C with a maximum of 5 mA per gel with a sequence of voltage set: constant voltage of 50 V (30 min), 100 V (30 min), and 150 V (30 min); thereafter, cathode buffer was renewed and only the 0.00002% (w/V) Serva Blue G was applied, and the separation continued at 200 V (approximately 2 h). NAD(P)H dehydrogenase in-gel activity of BN PAGE separated complexes was detected according to Quiles et al. (2000) in 50 mM potassium phosphate (pH 8.0), 1 mM Na_2_EDTA, 0.2 mM NAD(P)H, and 0.5 mg ml^−1^ Nitro Blue Tetrazolium. Colour development was achieved at 30°C in darkness. Gels were scanned using an Epson Perfection V750 PRO scanner. Peak detection based on the density of bands was carried out using Phoretix. Identification of bands was performed based on the second-dimension polypeptide patterns according to Basa et al. (2014) and Sárvári et al. (2022).

### 2.15 Ferric chelate reductase assay

Ferric chelate reductase (EC 1.16.1.9) activity was measured according to Solti et al. (2014a) with modifications according to Sági-Kazár et al. (2021). Plastidial inner envelope membrane fractions equivalent to 2–3 μg total protein suspended in TE buffer containing 0.2 M sucrose were mixed with equal volumes of 10 mM NADPH and 10 mM FAD solutions, and vesicles were loaded with these substances by using one freeze–thaw cycle (20/0°C), ensuring that only right-side-out envelope membrane vesicles could participate in the FCR reactions. NADPH and FAD-enclosing envelope vesicle suspensions were diluted by a HEPES-BPDS buffer to a final concentration of 50 mM HEPES-KOH (pH 7.0), 330 mM sorbitol, 2 mM EDTA, 2 mM MgCl_2_, 300 μM BPDS, 100 μM FAD, and 100 μM NADPH, and the envelope vesicles equivalent to 2–3 μg total protein. The reaction was initiated by adding Fe(III)-EDTA to the mixture. To eliminate background reactions of external NADPH and Fe(III)-EDTA, a reaction without the presence of FAD and NADPH was measured. While FAD and NADPH is present both in the vesicles and the reaction mixture, Fe(III)-EDTA and BPDS were absent from the vesicles; thus, FCR reaction could only be mediated by right-side-out vesicles, while in the background reaction, with the lack of FAD, even right-side-out vesicles could not mediate FCR activity. FCR reaction was monitored using a UV-VIS 2600 spectrophotometer mounted with Super Micro Black Cells (Shimadzu, Japan) at 535 nm for 20 min. Reaction rate was calculated from the linear phase of the absorption increase at 535 nm using an absorption coefficient of 22.14 mM^−1^ cm^−1^ (Smith et al., 1952).

### 2.16 Statistical analysis

The experiments were repeated on eight independent biological samples. XRF imaging was repeated 3 × 3 (ROI × biological samples) per leaf group. Element analysis was performed on three biological replicates. Chl content of the leaves and plastids was measured in 3 × 8 (technical × biological) repetitions. For relative transcript abundance analysis, samples were processed in three technical replicates on five biological parallels. Chl fluorescence lifetime measurements were performed in 5 × 8 (technical × biological) replicates. Chl *a* fluorescence measurements were repeated on five biological samples per leaf group. Chloroplast Fe uptake was performed in triplicates in five independent biological repetitions. To compare the variance between leaf groups, one-way ANOVAs with Tukey–Kramer *post-hoc* tests were performed on data using GraphPad Prism v.9.2 (GraphPad software Inc., USA). NADPH content was determined in 3 × 5 (technical × biological) replicates, and statistical differences were calculated using Student’s *t*-test. FCR assays were performed in 3 × 4 (technical × biological) repetitions. Data points were fitted with Boltzmann’s function in Origin v6.01 (Origin Lab Co., Northampton, MA, United States) and *v*_max_ and *K*_m_ values were calculated. Differences were compared using Student’s *t*-test. Transcript expression patterns were also compared with a Spearman correlation test. The term “significantly different” refers to a similarity of samples of *p* < 0.05.

## 3 Results

### 3.1 Degree of leaf etiolation

**Figure 1:**
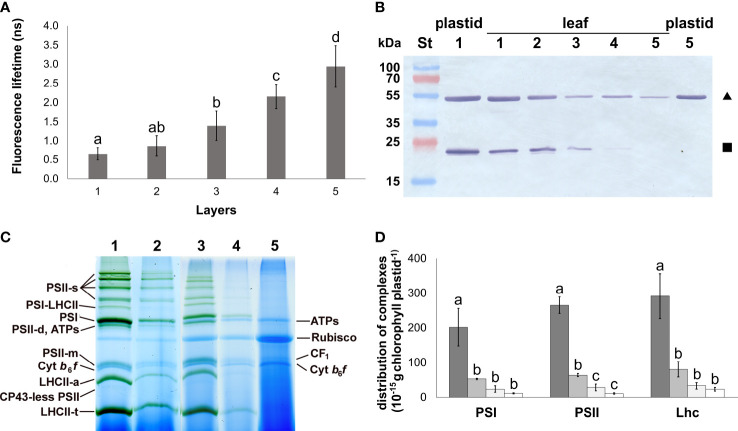
Photosynthetic development of different leaf layers of Savoy cabbage. **(A)** Fluorescence lifetimes of isolated plastids from different leaf layers. Differences were compared using one-way ANOVA with Tukey–Kramer *post-hoc* test [*p* < 0.05; *n* = 8 × 5 (biological × technical)]. Letters indicate groups with significant difference. **(B)** Combined immunoblot against Rubisco large subunit (RbcL, triangle) and Lhcb (square). Numbers 1–5 represent layers 1–5. **(C)** BN-PAGE pattern of the main plastid complexes isolated from different leaf layers (1–5), solubilised using 1% (w/V) *β*-DM plus 1% (w/V) digitonin, and separated in 4.5%–12% gel gradients. PS: photosystem; LHCII-a: Chl–protein (CP) 29 + CP24 + LHCII-t; ATPs: ATP synthase; Cyt: cytochrome; s: supercomplex; t: trimer; d: dimer; m: monomer, ribulose bisphosphate carboxylase oxygenase: Rubisco. Lanes 1–5 represent samples of Layers 1–5, respectively. **(D)** Amount of the main CPs in plastids of the different layers. They were not detected in Layer 5. Columns represent samples from Layer 1 (dark gray) to Layer 4 (open). Differences were compared using one-way ANOVA with Tukey–Kramer *post-hoc* test [*p* < 0.05; *n* = 3×5 (biological × technical)]. Letters indicate groups with significant difference. Error bars represent ± SD values.

The degree of etiolation in the separated leaf layers was determined based on the accumulation and function of photosynthetic pigments. Total Chl *a+b* content decreased significantly towards the innermost layers, while the Chl *a/b* ratio remained relatively stable, increasing significantly in Layer 4 (**Table 2**). Total Chl content and Chl *a*/*b* ratio were also applied to validate the isolated plastid samples to avoid an overrepresentation of any developmental stages of plastids in the isolates. In both leaves and isolated plastids of Layer 5, Chl content remained below our detection limit (**Table 2**). The intactness of plastids in the isolates was approved by the RbcL/apoLHCII ratio based on the comparison with intact leaves (**Figure 1B**). While this method is primarily limited to chloro- and etio-chloroplasts since it requires the presence of both markers, the average intactness of these isolates was found to be 85%–90%. Plastids isolated from the outer layers were slightly more vulnerable, likely due to their higher starch content.

**Table 2:** Physiological characterisation of the leaves and isolated plastids.

Layer	Leaf				Plastid	
	F_v_/*F*_m_	Φ_NF_	Chl *a*+*b*	Chl *a*/*b*	Chl *a*+*b*	Chl *a*/*b*
			µg^−1^ f.w.		10^-6^ ng plastid^−1^	
1	0.835 ± 0.009^a^	0.004 ± 0.005^a^	926.5 ± 79.3^a^	2.75 ± 0.19^a^	758.0 ± 117.3^a^	2.86 ± 0.07^a^
2	0.739 ± 0.025^ab^	0.100 ± 0.021^ab^	309.5 ± 54.5^b^	2.99 ± 0.12^a^	195.4 ± 21.4^b^	2.89 ± 0.20^a^
3	0.617 ± 0.079^bc^	0.269 ± 0.070^bc^	96.3 ± 12.6^c^	3.05 ± 0.13^ab^	84.6 ± 31.9^c^	2.99 ± 0.25^a^
4	0.530 ± 0.070^c^	0.383 ± 0.221^c^	38.7 ± 16.6^c^	3.58 ± 0.58^b^	44.9 ± 9.4^c^	3.47 ± 0.15^b^
5	0.492 ± 0.110^c^	0.385 ± 0.121^c^	nd	nd	nd	nd

Layers numbered from 1 to 5 represent outer to inner layers; nd, not detected. Errors are represented as ± SD. Differences were compared using one-way ANOVAs with Tukey–Kramer *post-hoc* tests [*p* < 0.05, *n* = 5 × 1; 5 × 1; 8 × 3; 8 × 3; 8 × 3; 8 × 3 (biological × technical) for each parameter, respectively]. Results of the statistical analyses are indicated by superscript letters for each dataset.

The status of the photosynthetic apparatus in the leaves was characterised by the maximum quantum efficiency of PSII reaction centres (*F*_v_/*F*_m_) and the proportion of excitation energy quenching of the non-functioning reaction centres (Φ_NF_) of the PSII reaction centres. In the separated leaf layers, a gradual decrease in the *F*_v_/*F*_m_ and, in parallel, an increased Φ_NF_ were measured (**Table 2**). The function of Chls referring to the operation of the photosynthetic machinery in the isolated plastids was characterised using fluorescence lifetime measurements. The Chl fluorescence lifetime showed a gradual increase among the plastid suspensions isolated from the light exposed towards the innermost leaf layers (**Figure 1A**). The fluorescence lifetime measured on the plastid suspensions isolated from the innermost Layer 5 was approximately half of that of the isolated Chls originating from Layer 1 in an acetone solution (**Supplementary Figure 3**).

The composition of the main complexes present in the isolated plastids from each leaf layer was analysed using 2D BN/SDS-PAGE (**Figure 1**; **Supplementary Figure 4**). Bands of low mobility in BN-PAGE were generally considered as PSII supercomplexes (PSII-s). PSI core monomers together with LHCI were found in PSI and PSI-LHCII bands, the latter also binding LHCII trimers (LHCII-t). ATP synthase complexes (ATPs) showed similar mobility in BN-PAGE to the PSII dimers (PSII-d). Components of PSII were also present in PSII monomer (PSII-m), and CP43-less PSII bands. Free Lhc complexes occurred as LHCII assembly (LHCII-a containing CP29, CP24, and LHCII-t), LHCII-t, and Lhc-m band components. The cytochrome *b_6_/f* dimers (Cyt *b_6_/f*-d) ran below the PSII-m band. The solubilised coupling factor (CF_1_) was present above the PSII-m band. Rubisco band appeared between ATPs and CF_1_. All these bands were detected in Layers 1–4; however, the amount of all chlorophyll–protein complexes in the plastid inner membranes strongly decreased towards the inner layers. The most significant difference was found between Layers 1 and 2 (**Figure 1D**). In Layer 5, only ATPs, Rubisco, CF_1_, and Cyt *b_6_/f*-d complexes were detected (**Supplementary Figure 4A**). The PSI/PSII ratio slightly increased towards Layer 4, while no significant changes were found in the LHCII/PSII ratio among the leaf layers (**Supplementary Figure 4B**). The composition of thylakoid complexes was nearly identical in the two outermost layers. In Layer 3 and Layer 4, the supercomplex organisation of thylakoid complexes was less predominant (**Supplementary Figures 4C–E**).

**Figure 2:**
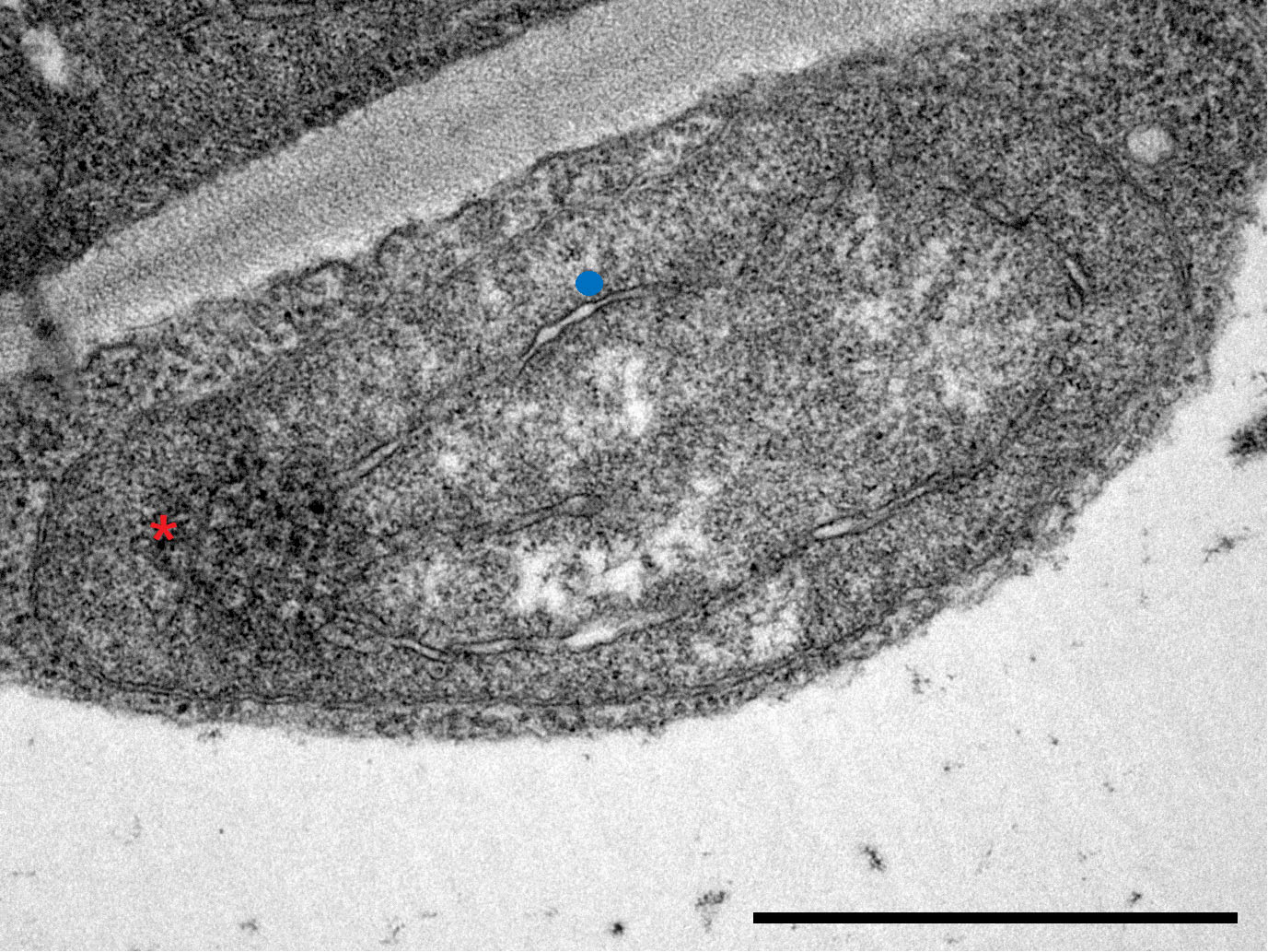
Transmission electron micrograph of a plastid in innermost, Layer 5 leaves of Savoy cabbage with both prolamellar body (red asterisk) and prothylakoids (blue dot). Bar is equal to 1 µm.

In terms of plastid ultrastructure, Layer 5 plastids (**Figure 2**) exhibited densely stained structures identified as prolamellar body (PLB), while only a few internal membrane lamellae were observed, which were identified as prothylakoids. Ferritin was not detected in these plastids, and starch accumulation was also not typical in these plastids.

### 3.2 Fe content and distribution

**Figure 3:**
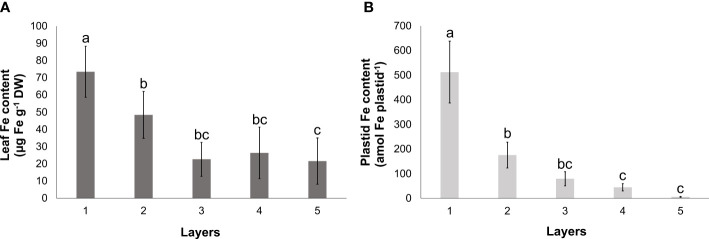
Fe content of leaves **(A)** and isolated plastids **(B)** of different leaf layers of Savoy cabbage. To compare differences, one-way ANOVA was performed with Tukey–Kramer *post-hoc* test **(A)** [*p* < 0.05; *n* = 3 × 3 (biological × technical)], **(B)** [*p* < 0.05; *n* = 5 × 3 (biological × technical)]. Letters indicate groups with significant difference. Error bars represent ± SD values.

Towards the inner leaf layers, the Fe content of leaves showed a gradual decrease. Similar to the changes in the Chl *a*+*b* content, a significant decrease was measured in leaf Fe content between Layers 1, 2, and 3, whereas the difference in the foliar Fe content among the inner leaf layers (Layers 3, 4, and 5) was not significant (**Figure 3A**). The Fe content of the plastids isolated from the leaf layers also exhibited a gradual decline from the outermost towards the inner layers. The Fe content in the innermost two layers (Layers 4 and 5) was found to be less than one-twentieth of that in the developed chloroplasts that were isolated from Layer 1 (**Figure 3B**).

**Figure 4:**
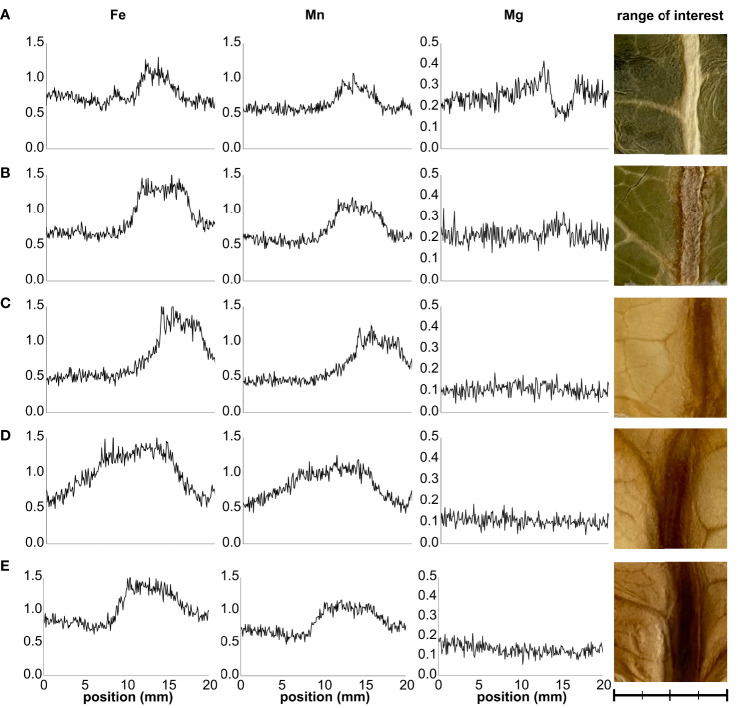
Representative distribution sections of Fe Kα_1_, Mn Kα_1_, and Mg Kα_1_ signals across the leaves of Layers 1 through 5 **(A–E)** of Savoy cabbage. Distribution maps represent cumulative signal intensities by pixel columns in the selected range of interest areas. Scale bar is equal to 20 mm with 5-mm minor intervals.

The Fe distribution across the leaf lamina was examined by XRF imaging (**Figure 4**). Layer 1 leaves exhibited a noticeable difference in the Fe Kα_1_ signal distribution between veinal and interveinal regions, with the former displaying higher Fe Kα_1_ emission (**Figure 4A**). This difference between the cumulative signal intensity of veinal to interveinal regions gradually increased towards Layer 3, but decreased from Layer 3 to Layer 5 significantly (**Figures 4B–E**; **Supplementary Figure 5**). Differences between the cumulative Mn Kα_1_ signal of veinal and interveinal regions changed in a similar tendency. To verify the Fe and Mn Kα_1_ signal distribution, Mg Kα_1_ signal was used as a reference. In Layer 1 leaves, veinal regions displayed low Mg Kα_1_ signal, while interveinal regions showed high emission. The difference in the Mg Kα_1_ signal between veinal and interveinal regions disappeared towards the inner leaf layers.

### 3.3 Relative transcript abundance of the plastid Fe homeostasis elements

**Figure 5:**
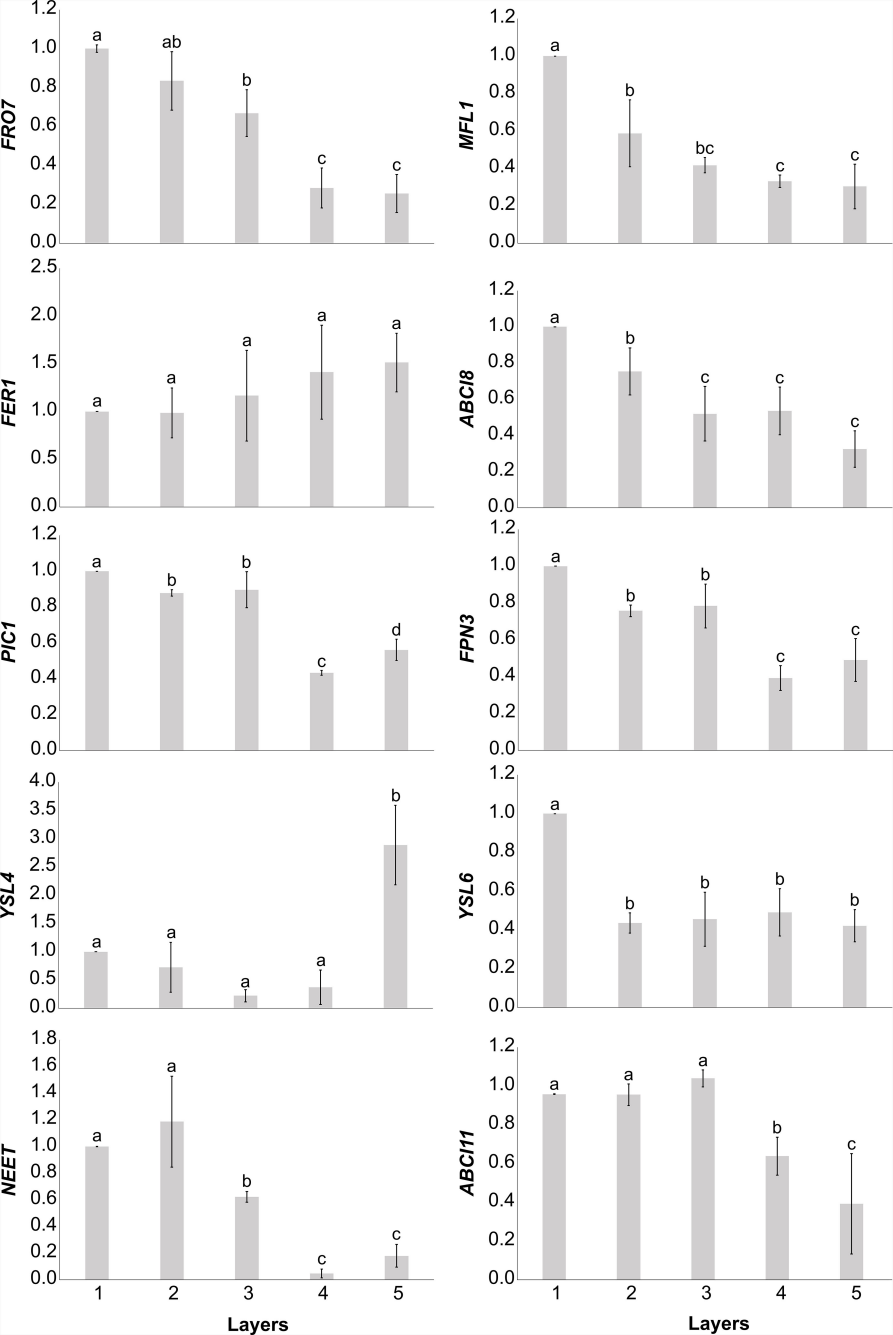
Normalised relative transcript abundance of Fe homeostasis elements in plastids of different leaf layers of Savoy cabbage. To compare differences, one-way ANOVAs were performed with Tukey–Kramer *post-hoc* tests on the individual genes of interest [*p* < 0.05, *n* = 5 × 3 (biological × technical)]. Letters indicate groups with significant difference. Error bars represent ± SD values.

The relative transcript abundance of selected Fe uptake and homeostasis elements of plastids was analysed (**Figure 5**). With the exception of *YSL4* and *FER1*, the relative transcript abundance of all studied genes of interest (GOIs) decreased from the outermost Layer 1 leaves towards the inner leaf layers. The relative transcript abundance of *FRO7*, *PIC1*, *FPN3, NEET*, and *ABCI11* proved to be more similar in Layers 1, 2, and 3 than in Layers 4 and 5, whereas that of *MFL1* and *YSL6* was distinct in Layer 1 but rather similar in the inner layers. Although there were no significant differences in the relative transcript abundance of *FER1* among the layers, a tendentious increase was measured towards the inner leaf layers. The relative transcript abundance of *YSL4*, on the other hand, was low in the outer leaf layers but significantly increased in Layer 5.

**Figure 6:**
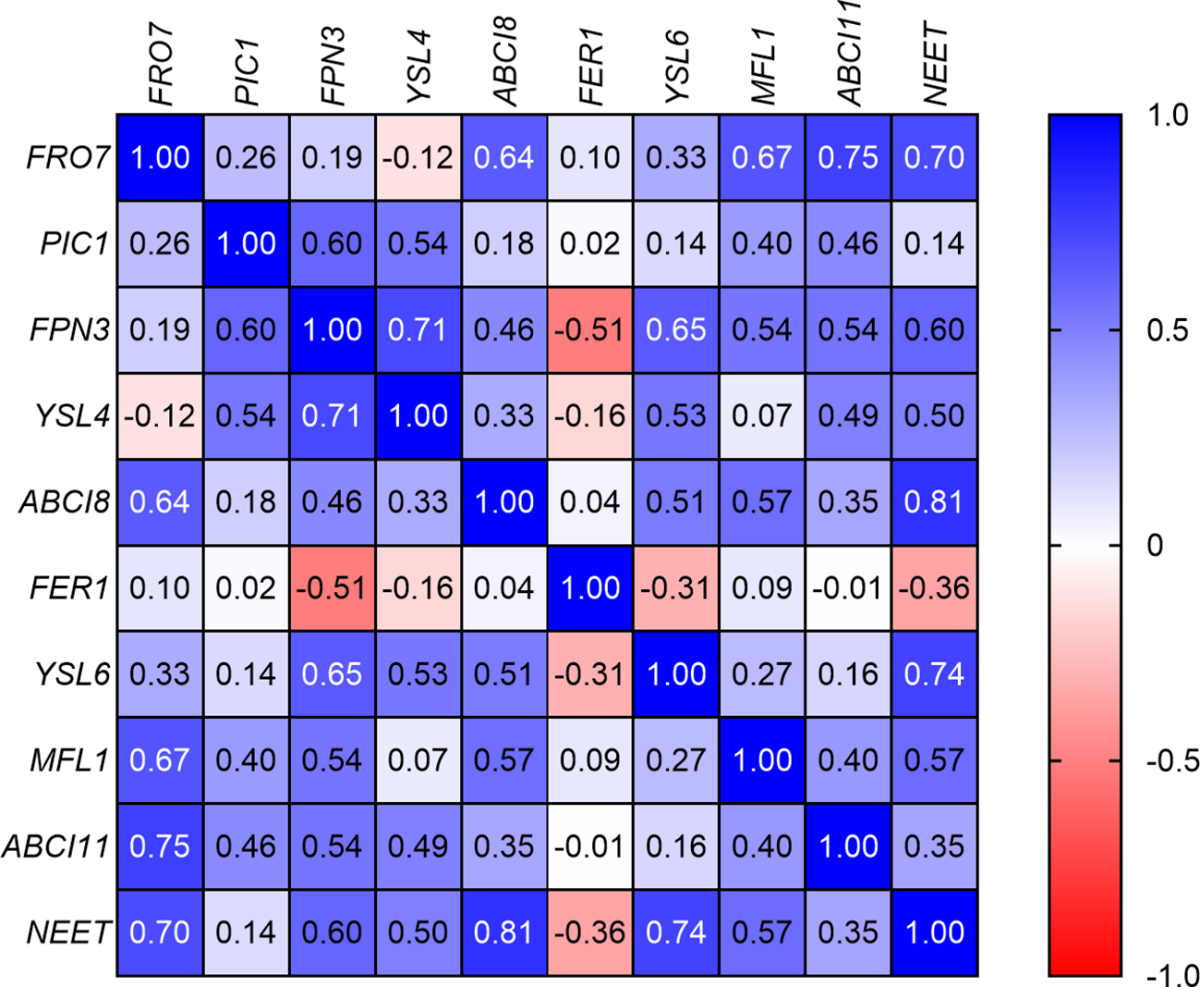
Correlation analysis of the relative transcript abundances of the genes of interest according to Spearman’s test (if −0.5 > *r* < 0.5, *p* < 0.05).

The correlation analysis of the GOIs (**Figure 6**) revealed that, with some exceptions, there was a significant positive correlation between Fe uptake and homeostasis elements such as *FRO7*, *ABCI8*, *MFL1*, *ABCI11*, and *NEET*. In addition, the relative transcript abundance of *NEET* showed significant positive correlation with that of *FPN3* and *YSL6*. On the other hand, the relative transcript abundance of *PIC1* displayed significant positive correlation with only *FPN3* and *YSL4*. Indeed, all three of these elements show a decreasing relative transcript abundance in Layers 1 through 4; this change is not significant in the case of *YSL4*. *FER1* showed no real correlation with other elements, while the only significant negative correlation was found between *FER1* and *FPN3*.

### 3.4 Plastidial Fe uptake, NADP(H) pool, and FCR activity

**Figure 7:**
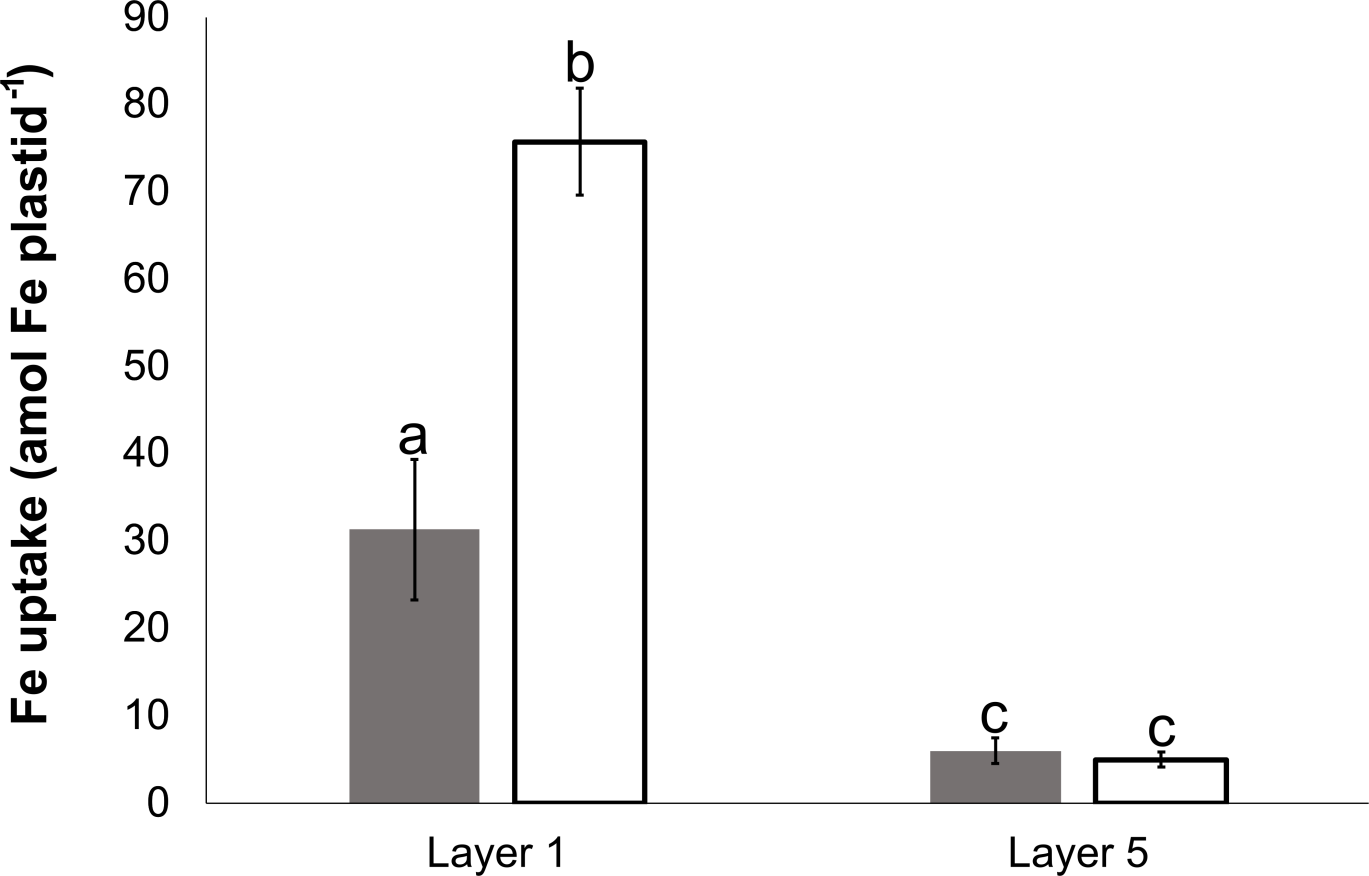
Fe uptake of plastids, isolated from Layer 1 and Layer 5 leaves, in darkness (dark gray) and on light (open) at 100 μM Fe(III)-citrate concentration. To compare differences within plastid suspensions, one-way ANOVA was performed with Tukey–Kramer *post-hoc* tests [*p* < 0.05; *n* = 4 × 3 (biological × technical)]. Letters indicate groups with significant difference. Error bars represent ± SD values.

The Fe uptake of plastids isolated from Layer 1 and Layer 5 was compared (**Figure 7**). Plastids isolated from Layer 1 leaves (**Figure 7A**) took up Fe both under dark and light conditions; however, the uptake remained significantly lower in the dark. In comparison, the Fe uptake in plastids obtained from Layer 5 leaves (**Figure 7B**) was consistently lower under both conditions.

The NADP(H) pool in Layer 1 and Layer 5 plastids [26.3 ± 3.24 and 23.04 ± 5.06 amol NADP(H) plastid^−1^, respectively] showed no difference (according to Student’s *t*-test, the difference is not significant, *p* > 0.05). NADPH dehydrogenase activity was detected in both Layer 1 and Layer 5 plastids using activity staining of BN-PAGE separated protein complexes (**Supplementary Figure 6**).

**Figure 8:**
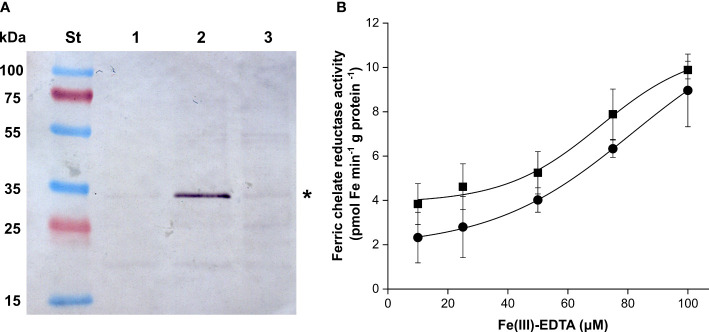
Detection chloroplast IEP37 (asterisk) **(A)**; lanes are as follows: St—Fermentas Page Ruler Pre-stained Protein SM0671 (Thermo-Fisher Scientific) pre-stained molecular weight standard, 1—mixed envelope fraction of Layer 5 plastids, 2—inner envelope fraction of Layer 5 plastids, 3—inner membrane fraction of Layer 5 plastids. **(B)** FCR activity of inner envelope fractions of Layer 1 (square) and Layer 5 (round) in the function of the applied Fe concentration. Error bars represent ± SD values.

Plastid suspensions from Layer 1 and Layer 5 were used to isolate IE vesicles. The identity of the isolated membrane fractions was verified by immunoblot against chloroplast IEP37 (**Figure 8A**). Approved IE fractions were subjected to FCR assay at multiple Fe concentrations. FCR activity was measurable in both types of samples (**Figure 8B**). The *v*_max_ values were 10.84 ± 1.39 and 12.2 ± 0.61 pmol Fe min^−1^ g protein^−1^ (no significant difference according to Student’s *t*-test, *p* > 0.05) and *K*_m_ values were 70.77 ± 8.62 and 81.49 ± 2.48 μM Fe-EDTA (significant difference according to Student’s *t*-test, *p* = 0.0475) for Layer 1 and Layer 5, respectively.

## 4 Discussion

### 4.1 Etiolation status of Savoy cabbage leaf layers

A gradual increase in etiolation was observed in the inner leaf layers of Savoy cabbage head, with Layer 5 leaves being considered completely etiolated. These leaves developed naturally in the complete absence of light and serve as a natural example of the etiolation syndrome (Solymosi et al., 2004; Kruk, 2005; Solymosi and Schoefs, 2010). The decreasing penetration of environmental light was reflected by the increasing fluorescence lifetimes, along with the increasing relative amount of Lhc and PSII monomers together with the decrease in the relative amount of PSII supercomplexes. Fluorescence lifetime measurements provide fundamental information on the state and operation of LHCs as previously done on isolated LHC complexes, thylakoid membranes, and single-cell algae (Dunn et al., 1994; Natali et al., 2016; Tutkus et al., 2021). Plastids isolated from the outer layers exhibited a fast fluorescence decay, suggesting that the excitation energy transfer towards the reaction centres and the structure of the antennae is intact. However, plastids in Layer 5 were heavily impacted, with no detectable Chl content or major Chl–protein complexes present in these leaves. Even a single leaf layer had a strong light filtering effect accounting for a significant drop in the total Chl content and in the amount of major Chl–protein complexes as seen in the leaves of Layer 2 similarly to previous observations (Solymosi et al., 2004; Kruk, 2005; Solymosi et al., 2012). However, despite the decrease in transmitted light towards Layers 2 and 3, the relatively stable Chl *a/b* ratio suggests that the photomorphogenesis of the leaves was not significantly impacted. Therefore, plastids from Layers 1, 2, and 3 may be considered as chloroplasts. In contrast, plastids isolated from Layer 5 exhibited no detectable Chl–protein complexes and Chl. Thylakoid complexes were limited to ATP synthase and Cyt *b_6_/f* complexes, with the presence of prothylakoids and a large PLB detected by TEM, further supporting the classification of Layer 5 leaves and plastids as etiolated. Additionally, no structures associated with ferritin complexes were found in Layer 5 etioplasts (Sági-Kazár et al., 2022). Considering the slightly increased expression of *FER1* towards the inner leaf layers, the presence of ferritin (apo)protein cannot be ruled out, although the abundance of ferritin complexes remained below the detection limit. Nevertheless, it was concluded that the inner leaves of Savoy cabbage are not specialised for storage, in contrast to the inner leaves of white cabbage (*B. oleracea* var. *capitata*) that were found to accumulate a considerable amount of ferritin (Solymosi et al., 2004).

### 4.2 Etiolation affects the Fe homeostasis

Fe, in the form of cofactors, is required for various proteins, while its homeostasis in leaves is primarily determined by its transport towards the (chloro)plasts, which represent the major Fe sink in leaves (Hantzis et al., 2018; Schmidt et al., 2020). Fe reaches the leaves primarily as Fe(III)-citrate through the xylem, which with other Fe(III)-carboxylates is likely to infiltrate the mesophyll apoplast (Sági-Kazár et al., 2022). Phloem-based Fe transport mainly supports tissues that are not reached by differentiated xylem vessels (Klatte et al., 2009; Schuler et al., 2012; Sági-Kazár et al., 2022). Roschzttardtz et al. (2013) demonstrated that Fe mainly accumulates in the parenchyma cells of the vasculature region in *Arabidopsis* rosette leaves. Also studying *fer1,3,4* triple mutants under Fe excess, the Fe accumulation in the vascular parenchyma was concluded as ferritin complexes. Therefore, the higher Fe Kα_1_ signal detected by XRF imaging in our study likely originated from the Fe signal in the vascular elements along with Fe retention in the vascular parenchyma. The increased difference between the signal intensity in veinal and interveinal regions in Layer 3, together with the decreased accumulation of pigment–protein complexes in this leaf layer, indicates that a decrease in the light intensity leads to a retention of Fe along the veins, most probably in the vascular parenchyma. The similar Mn Kα_1_ but antiparallel Mg Kα_1_ signal patterns suggest that the thicker anatomical structures at the vascular regions are not responsible for the higher Fe Kα_1_ signal. Although decreased compared to that of Layer 3, the difference between the intensity of the Fe Kα_1_ signal remained substantial between the veinal and interveinal region in etiolated leaves, indicating that the Fe retention in parenchyma cells of the vasculature region is supposed to be predominant and affects etiolated leaves, too. Consequently, the Fe distribution in the etiolated leaves of Savoy cabbage head shares many similarities to that of *Arabidopsis* cotyledons (Roschzttardtz et al., 2009).

Even though there was a declining tendency in the Fe content of isolated plastids as the degree of etiolation increased, Fe was still present even in the plastids of the fully etiolated Layer 5 leaves. Although photosynthetic machinery, especially PSI complexes, has the highest Fe requirements in plants, multiple other plastidial proteins, including those related to photosynthesis such as PETA, PETB, and PETC chains of the Cyt *b_6_/f* complex, NDHI and NDHK of the NDH complex, ferredoxins, chlorophyll cyclase CRD1/CHl27, 7-hydroxymethyl chlorophyll *a* reductase, as well as those primarily unrelated to photosynthesis such as Fe superoxide dismutase, ascorbate peroxidases, lipopoxygenases, adenosine 5′-phosphosulfate reductase, sulfite reductase, plastidial nitrate reductase, and glutamine:oxoglutarate aminotransferase, require Fe cofactors for proper functioning (Hantzis et al., 2018; Lu, 2018; Kroh and Pilon, 2020; Przybyla-Toscano et al., 2021). Unlike *Nicotiana clevelandii* × *N. glutinosa* cotyledons (Sprey et al., 1977), Fe accumulation in ferritin was not detected in the etioplasts of Savoy cabbage; thus, the biosynthesis of Fe-containing proteins relies on the continuous import of Fe into the plastids. Protoheme production in barley (*Hordeum vulgare*) plastids was found to be light-triggered (Ameen and Miller, 1984). However, Ferrochelatase (FC) 1 (also named Genomes Uncoupled; GUN6) is present in etioplasts (Little and Jones, 1976; Jarvis and Lopez-Juez, 2013). Plastidial heme biosynthesis is involved in the plastid-to-nucleus retrograde signalling (Shimizu et al., 2019): FC1 is responsible for the delivery of heme groups to heme proteins outside of the plastids (Richter et al., 2022). However, no dedicated heme transport protein was identified in the plastidial envelope membranes, which raises the question whether heme can be transported through hydrophobic channels such as inter-organellar membrane contact sites (Chambers et al., 2021; Richter et al., 2022). Additionally, plastids synthesise Fe-S clusters for both internal use and delivery to cytosolic proteins (Zandalinas et al., 2020). However, research on the biosynthesis of Fe-S clusters in non-photosynthetic plastids is limited (Balk and Lobréaux, 2005; Przybyla-Toscano et al., 2018). Nevertheless, the low but detectable relative transcript abundance of Fe-S cluster biosynthesis and processing machinery members *ATP-Binding Casette I* (*ABCI*) *6*, *ABCI8*, *ABCI9*, and *Glutaredoxin* (*GRXS*) *14* in non-green tissues (arabidopsis.org; Klepikova et al., 2016) suggests the essential role of plastidial Fe-S cluster biosynthesis in non-photosynthetic plastids. Similar to *Arabidopsis* transcriptomic data, we observed a moderate but notable expression level of *ABCI8* in etiolated leaves. In consequence, the ferrochelatase activity, the Fe-S cluster biosynthesis activity, and, to support them, the presence and continuous import of Fe into etioplasts and other non-photosynthetic plastids are essential. According to our results, the Fe content of the plastids increases significantly in parallel with the de-etiolation, but etioplasts already contain Fe, despite the absence of photosynthetic structures. Considering the biochemical activity and the need for Fe in plastids, this basic Fe content exists but is masked in photosynthetically active chloroplasts by the high Fe content of the photosynthetic machinery.

Chloroplasts of the photosynthetically active tissues take up Fe using a reduction-based mechanism (Jeong et al., 2008; Solti et al., 2012) where the reducing capacity utilised by FRO7 originates from the oxidation of NADPH (Solti et al., 2014a). Additionally, photoreduction of Fe(III)-citrate also contributes to Fe^2+^ generation (Jeong et al., 2008; Gracheva et al., 2022). The disruption of the NADPH production by photosynthesis inhibitors or by darkness generally hinders the Fe uptake of chloroplasts (Bughio et al., 1997; Solti et al., 2012). As a result, the Fe uptake of chloroplasts through FRO7-mediated, reduction-based mechanism heavily depends on the NADPH produced during photosynthesis. Mitochondria also operate a reduction-based Fe uptake including FRO3 and FRO8 (for review see: Sági-Kazár et al., 2022). In contrast to plastids, the localisation of mitochondrial FRO enzymes has not been clarified yet. TCA cycle in mitochondria of both photosynthetic and non-photosynthetic mesophyll cells generates NADH, driving the ATP synthesis and thus the energy homeostasis, but the contribution of TCA cycle to mitochondrial Fe uptake has not been tested so far. While photosynthetically active chloroplasts generate NADPH primarily through the photosynthetic electron transport chain (ferredoxin:NADP oxidoreductase; FNR) (Takahama et al., 1981), non-photosynthetic plastids generate NADPH solely by the oxidative pentose phosphate pathway (Kruger and von Schaewen, 2003). The existence of NADPH-based reducing power is essential for a series of metabolic functions in non-photosynthetic plastids (Neuhaus and Emes, 2000). Among others, enzymes of nitrogen and sulphur assimilation pathways have a high need for reducing power and are abundant in non-photosynthetic plastids (Daher et al., 2010; Grabsztunowicz et al., 2020). Additionally, the NADPH-dependent Thioredoxin Reductase C control system is utilised by non-photosynthetic plastids, which plays a crucial role in metabolic regulation, growth, and development of plant tissues (Ferrández et al., 2012; Cejudo et al., 2021). Our data highlight the significance of NADP(H) in non-photosynthetic plastids, as we observed no changes in the total NADP(H) pool size between Layer 1 and 5 plastids. Moreover, the detection of NDH complex activity in Layer 5 etioplasts is consistent with that observed in barley etioplasts (Guera et al., 2000), suggesting that Layer 5 etioplasts maintain an active NADP(H) metabolism. *In vitro* FCR activity of the isolated IE membrane vesicles of Layer 5 etioplasts, where the enzyme activity was driven by added NADPH, proved that etioplasts are capable of operating the reduction-based Fe uptake system. Nevertheless, the low Fe uptake of isolated etioplasts, together with the correlating and low expression of Fe uptake and homeostasis elements *FRO7*, *ABCI8*, *MFL1*, *ABCI11*, and *NEET*, indicates that the Fe acquisition and incorporation operates in a low activity mode compared to chloroplasts. Illumination did not trigger the Fe uptake of isolated etioplasts. Since these plastids lacked the functional photosynthetic machinery, it could not have contributed to the reduction of NADP^+^. Altogether, the Fe acquisition of etioplasts is low, which corresponds to the observed relative transcript abundance of multiple Fe homeostasis elements.

In contrast to the majority of plastidial Fe homeostasis elements, the relative transcript abundance of *YSL4* showed an increased level in Layer 5 leaves of Savoy cabbage. Divol et al. (2013) found that in *Arabidopsis*, *YSL4* expression is ubiquitous but low. In *ysl4ysl6* double mutants, under optimum Fe nutrition, plastids in the vascular region showed Fe accumulation; thus, YSL4 was postulated as a plastidial Fe transporter. However, Conte et al. (2013) argued that both YSL 4 and 6 are localised in the tonoplast and in the endoplasmic reticulum. Based on *pYSL:GUS* transgenic lines, YSL4 is present in leaves but the abundance is even higher in root tips, yet nearly absent in senescent tissues. Müller et al. (2019) reported that in *B. napus*, *YSL4* has a high expression in ripening siliques. In consequence, the role of YSL4 remained elusive. Recently, Kim et al. (2021) reported that the mutation of mitochondrial/chloroplast Fe exporter Ferroportin 3 resulted in an elevated relative transcript abundance of *YSL4*. Here, we also find an antiparallel change in the relative transcript amount of *FPN3* and *YSL4* in Layer 5 etiolated leaves. Considering the data that YSL4 can act as a Fe exporter, the increased *YSL4* expression with low *FPN3* transcript level indicates that the process and pathways of intracellular Fe recycling in etiolated tissues are distinct from those in green tissues.

## 5 Conclusion

The development of the photosynthetic machinery in green tissues requires a massive amount of Fe, which is supported by the reduction-based Fe acquisition mechanism of chloroplasts. However, this mechanism cannot operate in the same manner in non-photosynthetic plastids since it relies on NADPH produced by the photosynthetic electron transport chain. Etioplasts, which are mesophyll cell plastids that do not develop into chloroplasts due to the absence of light, offer a useful model to study Fe homeostasis in leaf plastids without the bias of the high Fe need of photosynthesis. While etioplasts are capable of Fe uptake, as Fe is also important as a cofactor of multiple plastidial metabolic pathways, the process is likely to rely on metabolically produced NADPH and occurs at a lower rate than in chloroplasts. Etioplasts of Savoy cabbage do not store a significant amount of ferritin, and their Fe acquisition operates in parallel to the formation of the photosynthetic machinery. This suggests that the accumulation of Fe in chloroplasts and the development of the photosynthetic apparatus are closely linked.

## Data availability statement

The original contributions presented in the study are included in the article/**Supplementary Material**. Further inquiries can be directed to the corresponding author.

## Author contributions

MS-K and ÁS designed and supervised the study. Chl *a* fluorescence induction was measured by ÁS. XRF imaging was performed by MS-K. Element content measurement was performed by MS-K and ZM. Chl lifetime measurements were performed by LI, AB, and SL. TEM analysis was done by KS. Bioinformatics, expression analysis, and the isolation of plastids and envelope membranes were performed by MS-K, BC, and CH. Fe uptake, FCR assays, and the NADP(H) pool were measured by MS-K and BC. Blue Native PAGE separations and Western blots were done by ÉS. MS-K and ÁS wrote the manuscript, and all authors critically reviewed it. All authors contributed to the article and approved the submitted version.
