# Supplementary material for: Iron uptake of etioplasts is independent from photosynthesis but applies the reduction-based strategy
Source: Front Plant Sci. 2023 Aug 11;14:1227811. doi: 10.3389/fpls.2023.1227811 (PMC10457162; doi:10.3389/fpls.2023.1227811)
Supplement: Supplementary file 1 [file DataSheet_1.pdf]

Supplementary Material

Iron uptake of etioplasts is independent from photosynthesis but applies the reduction based strategy

Máté Sági-Kazár, Éva Sárvári, Barnabás Cseh, Levente Illés, Zoltán May, Csaba Hegedűs, Attila Barócsi, Sándor Lenk, Katalin Solymosi, Ádám Solti*

*** Correspondence:** Ádám Solti: adam.solti@ttk.elte.hu

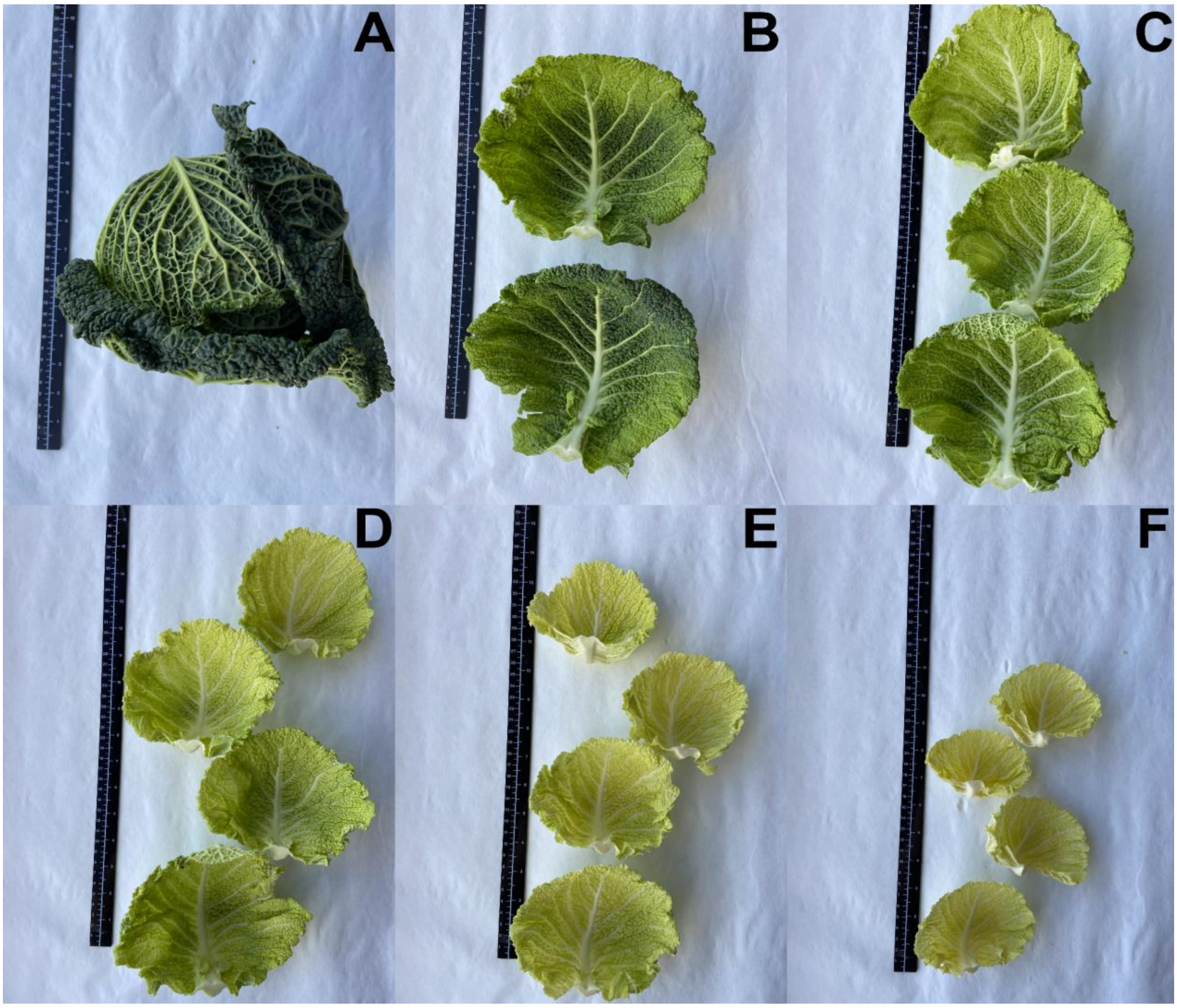

Supplementary Figure 1. Macroscopic appearance of Savoy cabbage (*Brassica oleracea* var. *sabauda* L.) leaf layers. (A) Medium sized Savoy cabbage head prior to separation. (B-F) Layers of leaves (1-5) separated based on whole head coverage.

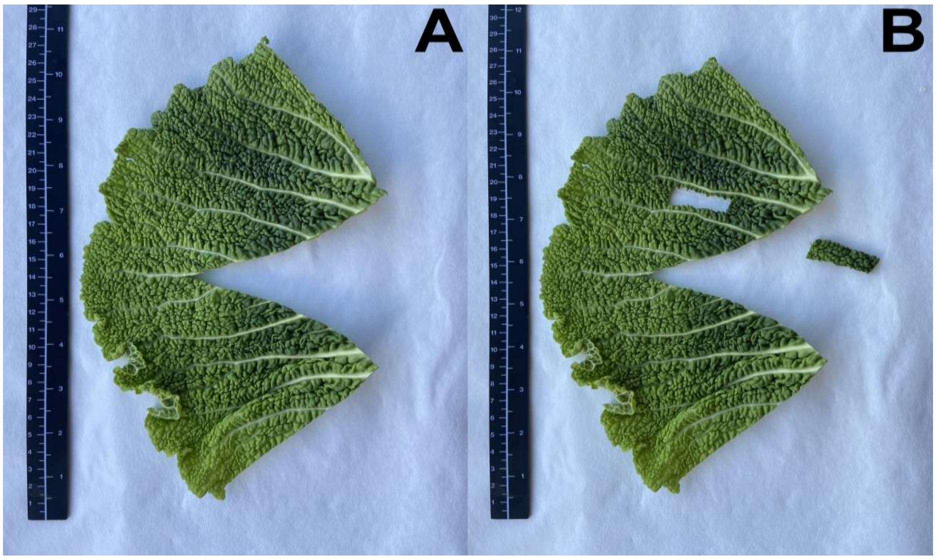

Supplementary Figure 2. The method of sample preparation: (A) leaf area used for isolation procedures; (B) position of sample collection for RNA extraction, ICP-MS, and Chl measurement.

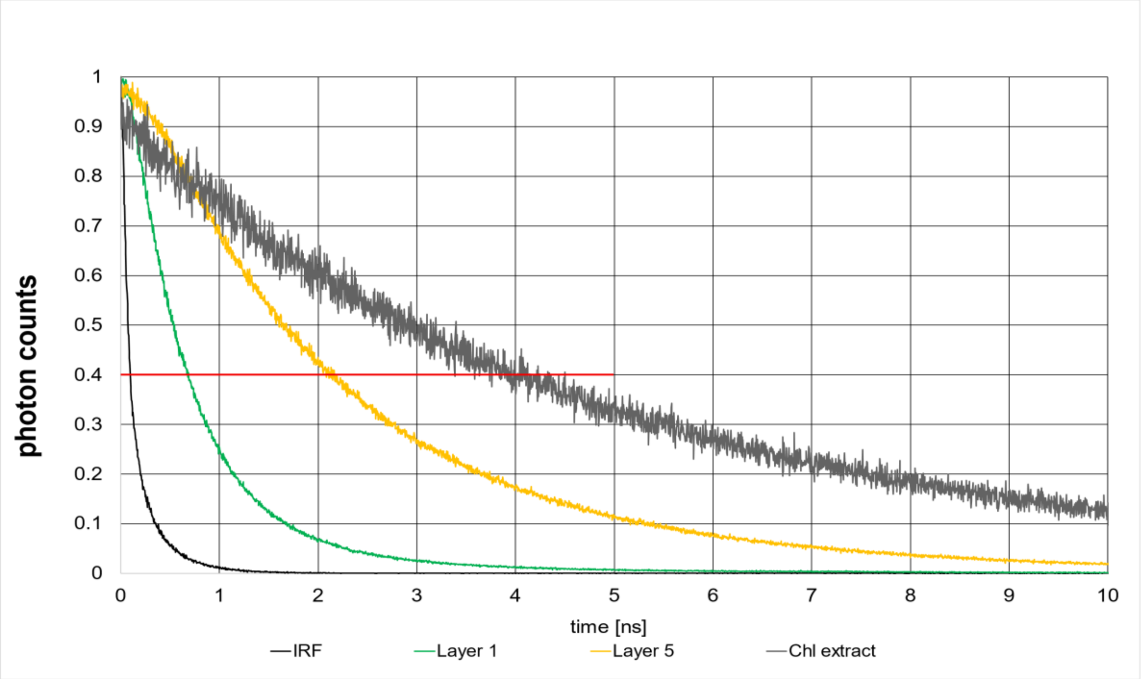

Supplementary Figure 3. Fluorescence decay kinetics of isolated Chls in acetone solution compared to plastids isolated from Layer 1 and Layer 5 (IRF – Instrument Response Function). Red line indicates the approximate fluorescence lifetimes.

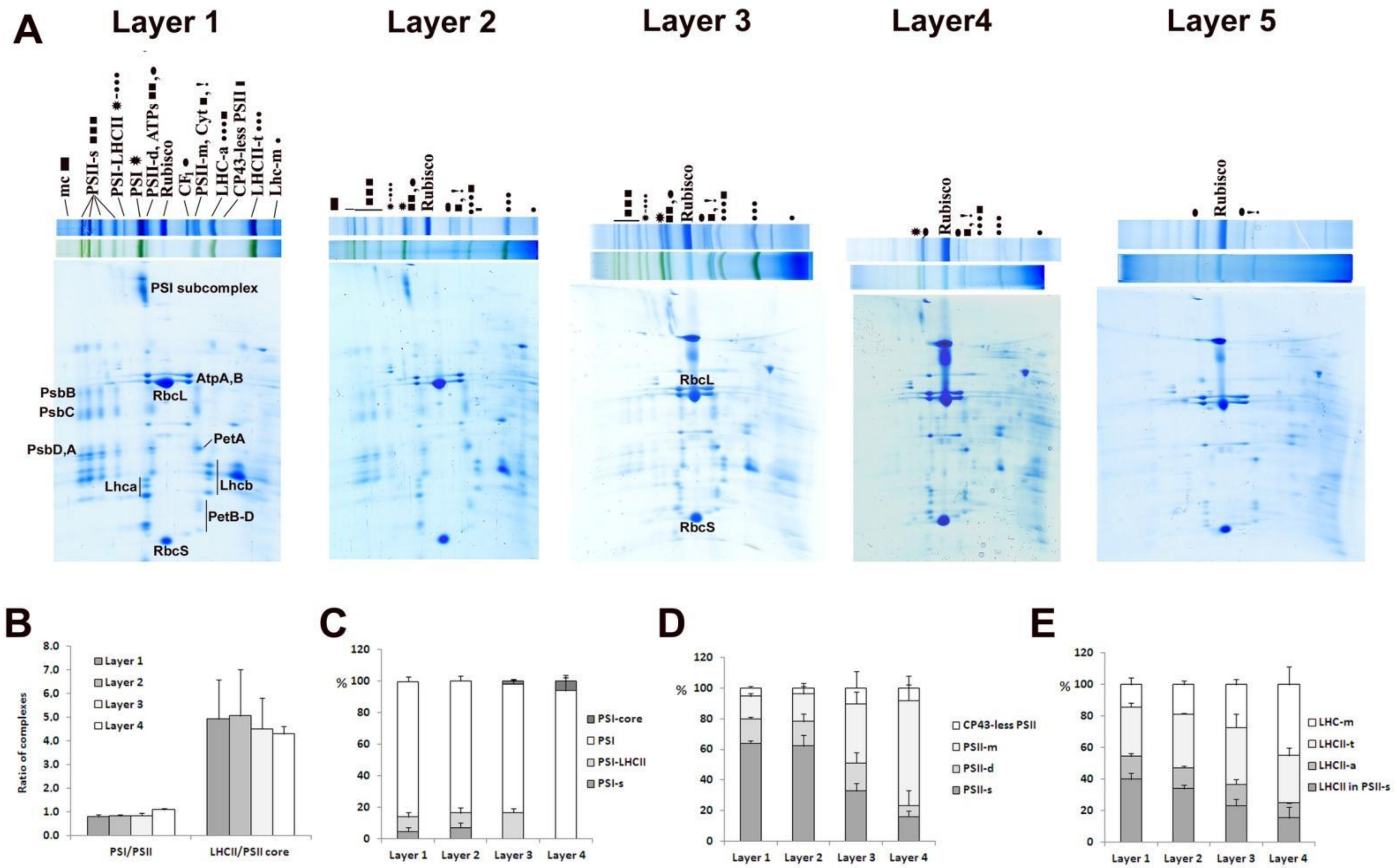
A
Layer 1
Layer 2
Layer 3
Layer4
Layer 5
mc
PSII-s
PSI-LHCII
PSI
PSII-d, ATPs
Rubisco
CF1
PSII-m, Cyt
LHC-a
CP43-less PSII
LHCII-t
Lhc-m
PSI subcomplex
AtpA,B
PsbB
RbcL
PsbC
PetA
PsbD,A
Lhca
Lhcb
PetB-D
RbcS
B
Ratio of complexes
Layer 1
Layer 2
Layer 3
Layer 4
PSI/PSII
LHCII/PSII core
C
%
PSI-core
PSI
PSI-LHCII
PSI-s
D
CP43-less PSII
PSII-m
PSII-d
PSII-s
E
LHC-m
LHCII-t
LHCII-a
LHCII in PSII-s

Supplementary Figure 4. Complex patterns and thylakoid organization in the different leaf layers. (A) Middle lanes: Blue Native PAGE pattern of plastids isolated from different leaf layers, solubilised using 1% (w/V) β-DM plus 1% (w/V) digitonin, and separated in 4.5-12% gel gradients. PS: photosytem; LHCII-a: CP29 + CP24 + LHCII-t; ATPs: ATP synthase; Cyt: cytochrome; mc: megacomplex; s: supercomplex; t: trimer; d: dimer; m: monomer, ribulose bisphosphate carboxylase oxygenase: Rubisco. Upper lanes: BN PAGE lanes stained with Coomassie. The staining of the complexes which contained only proteins were stronger than that of the pigment-protein complexes. Lower part: Polypeptide patterns of complex bands determined by 2ndD SDS PAGE. The polypeptides used to identify complexes are marked. (B) Ratios of the main chlorophyll-protein complexes. (C-E) Distribution of PSI, PSII, and Lhc among their different complex forms. Error bars represent ±SD values.

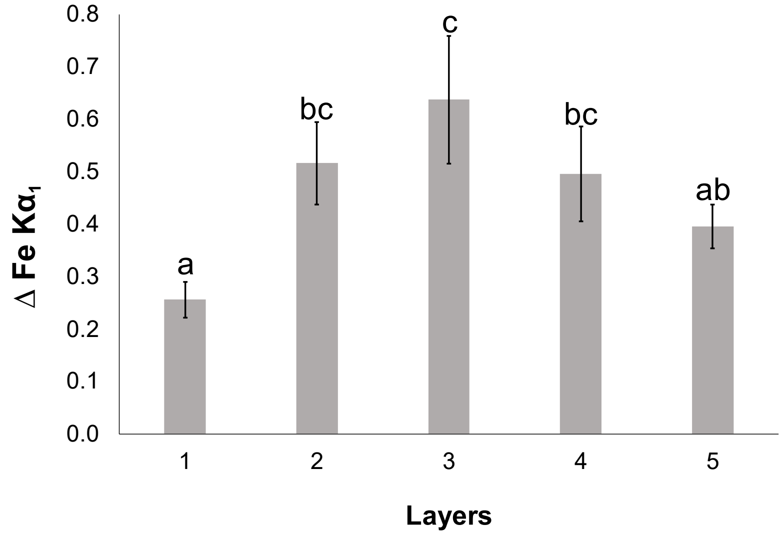

Supplementary Figure 5. Differences between the cumulative Fe Kα_1 signal intensities of corresponding venal and interveinal regions (Δ Fe Kα_1) of leaf layers of Savoy cabbage. Error bars represent ±SD values. Letters indicate groups with significant difference following one-way ANOVA with Tukey-Kramer *post hoc* test [$P < 0.05$, n=3×3 (biological×ROI)].

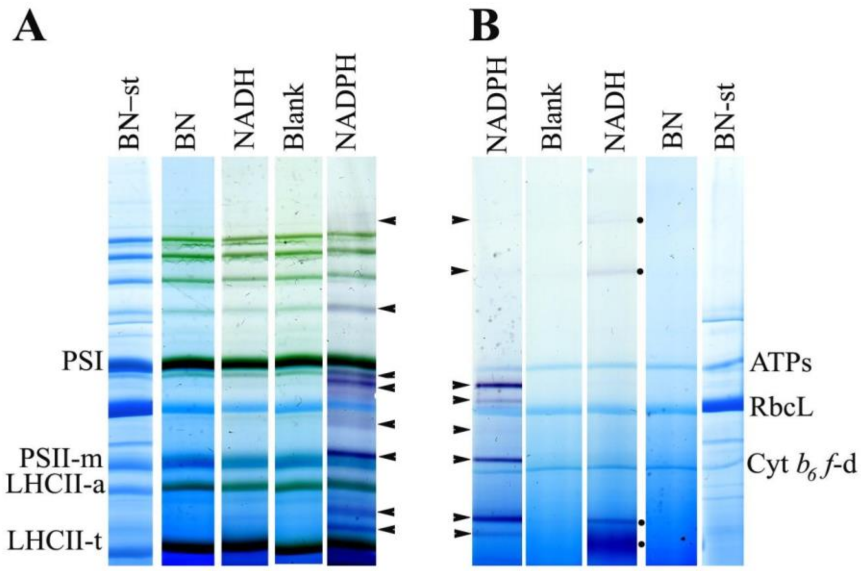

Supplementary Figure 6. NADH dehydrogenase-like activity in Layer 1 (A) and Layer 5 (B) BN-st: Coomassie stained bands of the complexes separated in the first dimension Blue Native PAGE. Bands indicating a specific staining in the presence of NADH, and NADPH are marked by points and arrowheads, respectively.
